# Association Between Dietary Live Microbes, Folate, and Fatigue: A Cross-Sectional Study in U.S. Older Adults

**DOI:** 10.1093/geroni/igaf122.2429

**Published:** 2025-12-31

**Authors:** Galya Bigman, Amber Kleckner, Elizabeth Dennis, John Sorkin, Alice Ryan

**Affiliations:** University of Maryland, Baltimore County, Baltimore, Maryland, United States; University of Maryland School of Nursing, Baltimore, Maryland, United States; University of Maryland School of Medicine, Baltimore, Maryland, United States; University of Maryland School of Nursing, Baltimore, Maryland, United States; University of Maryland School of Medicine, Baltimore, Maryland, United States

## Abstract

Fatigue is a common and debilitating condition in older adults, linked to chronic inflammation, gut-brain axis dysfunction, and impaired metabolic health. Live microbes in fermented foods and dietary fibers support short-chain fatty acid (SCFA) production, potentially alleviating fatigue by modulating these mechanisms and enhancing nutrient bioavailability. Folate, essential for energy metabolism, is associated with fatigue, and its bioavailability is influenced by the gut microbiome through live microbe intake. This study examines the relationship between live microbe intake and fatigue, with folate as a mediator. We analyzed data from 2,353 older U.S. adults (≥60 years) in NHANES (2021–2023). Fatigue, assessed via the PHQ-9, was categorized as present or absent. RBC folate, a biomarker of long-term status, was classified into tertiles. Dietary microbe intake, including probiotics, was derived from 24-hour recalls and categorized as Low (< 10^4^ CFU/g), Medium (10^4^–10^7^ CFU/g), or High (>10^7^ CFU/g). Covariates included demographics, BMI, comorbidities, lifestyle, and energy intake. Multivariable regression and structural equation modeling accounted for survey design. The mean age was 69.4 years (SD = 6.3), with 46.1% male. Live microbe intake was low (28.3%), medium (45.5%), or high (26.5%), and 15.4% reported fatigue. Higher microbe intake was linked to lower fatigue odds (OR = 0.58, p = 0.021) and increased folate levels (OR = 1.38, p = 0.015), with folate mediating 21.8% of the effect. While findings suggest dietary microbes may reduce fatigue via folate, causal inference requires further study.

